# A qualitative study on stress, coping strategies and feasibility of music intervention among women with cancer receiving chemotherapy during COVID-19 pandemic in Vietnam

**DOI:** 10.1038/s41598-023-27654-9

**Published:** 2023-01-11

**Authors:** Khanh T. Nguyen, Nhung T. H. Vu, Mai T. T. Tran, Carmen W. H. Chan

**Affiliations:** 1 The Nethersole School of Nursing, The Chinese University of Hong Kong, Shatin, NT, Hong Kong SAR China; 2 Nam Dinh University of Nursing, Nam Dinh, Vietnam

**Keywords:** Psychology, Health care, Oncology

## Abstract

Breast and gynaecological cancer (BGC) patients receiving chemotherapy may experience high levels of stress during the COVID-19 pandemic. Music interventions may be effective in lowering their stress levels. This study explored stressors, coping strategies, and the feasibility of music interventions among BGC patients in Vietnam. An exploratory qualitative study with individual face-to-face semi-structured interviews was conducted. A convenience sample of BGC patients receiving chemotherapy was recruited from the oncology centre of a public hospital in Vietnam. Twenty patients were interviewed with open-ended questions developed based on the transactional model of stress and coping to explore stress-causing factors and coping strategies and based on guidelines for music therapy practice to explore their music preferences and perceptions. Field notes and interview transcripts were analysed following the qualitative content analysis approach. Two stressor themes were identified: undesirable experiences during treatment and patients’ inability to fulfil their own roles and responsibilities. Our findings revealed a new coping strategy—self-realisation of responsibilities towards the family—that is not listed in the transactional model of stress and coping. Future psychological interventions for stress management among BGC patients should focus on raising the patients’ awareness of their values and responsibilities towards their families. Three categories of preferred music genres for stress reduction were identified: religious, softly melodic, and revolutionary music. The patients were aware of the positive effects of music and had different musical preferences. This study also explored the acceptance of music interventions and facilitators and barriers to implementing them among BGC patients in Vietnam. The findings suggest that before implementing music interventions, the musical preferences, religions, and beliefs of each individual should be considered to achieve desirable results. Music interventions for BGC patients receiving chemotherapy in Vietnam are feasible. Further intervention studies are needed to evaluate their effectiveness.

## Introduction

The entire cancer trajectory from diagnosis to treatment is a stressful experience for cancer patients, including those with breast and gynaecological cancer (BGC)^1^. They face a variety of stressors, such as the cost of therapy, changes in relationships, changes in body image, and side effects of treatment, that add to the psychological stress experienced by these patients^2^. The COVID-19 pandemic that peaked around April 2021 in Vietnam has been cited as an additional stress factor for cancer patients^3^. As the number of patients with COVID-19 increased rapidly, the government locked down major cities to prevent the spread of the pandemic. All patients, including BGC patients, faced difficulties in accessing medical facilities and services, which negatively affected their disease management and raised their stress levels^4^. People adopted various adaptive (positive) and maladaptive (negative) coping strategies to manage stress during the pandemic^5^. Among these, the six coping strategies commonly adopted by cancer patients are seeking information, seeking support and comfort, attributing causes to events, taking impulsive action, avoiding confrontation, and adopting active coping behaviour to reduce stress^2^. A survey on women with ovarian cancer during the pandemic showed that most participants used at least one adaptive coping strategy, while 35.8% used at least one maladaptive coping strategy^6^. Thus, more studies on supportive programmes to strengthen adaptive coping strategies and minimise maladaptive coping strategies in this population are necessary^5^.

Lazarus^7^ developed the transactional model of stress and coping (TMSC), which explains the cognitive processes, namely primary and secondary appraisals, used by people to evaluate a situation and identify stress. People experience stress if they identify a situation as harmful/loss inducing, threatening, or challenging but have inadequate resources to deal with it. Then, they may adopt appropriate coping strategies, such as problem-focused and emotion-focused coping, to overcome the situation^7^. Problem-focused ways of coping are more likely to be adopted when an appraisal can be done to modify the stressful situation^7^. Problem-focused coping strategies include active coping, planning, suppressing competing activities, restraint coping, and seeking instrumental social support^8^. In contrast, emotion-focused ways of coping aim to manage the emotions elicited by a situation rather than amend the situation itself^9^. Emotion-focused coping strategies include self-blame, distraction, religion, acceptance, and emotional support^7^.

Undergoing chemotherapy during the COVID-19 pandemic makes for a particularly stressful experience under circumstances that cannot be modified easily, and contributes to the suffering of BGC patients. Thus, emotion-focused coping may be more effective than problem-focused coping to manage stress among these patients. Among the emotion-focused coping strategies, music interventions have been found to be effective in reducing stress^10^ and may be an effective tool for improving cancer patients’ psychological and physical outcomes^11–13^. A previous systematic review on music interventions showed significantly reduced emotional distress, such as anxiety disorder, and improved quality of life among cancer patients receiving chemotherapy^14^. Music stimulates the nervous system, especially the hypothalamus and the limbic system, which control stress hormone release and emotional reactions^15^. A previous study demonstrated a significant decrease in the levels of cortisol (the main stress hormone) after music therapy in cancer patients^16^.

The goals of music interventions in healthcare are to manage symptoms, enhance quality of life, support physical and/or psychosocial function, and/or promote well-being (including spirituality)^17^. Music interventions are divided into two types based on methodology: music therapy, which is provided by a trained therapist using a variety of strategies, including music listening, song writing, improvisation, and lyric analysis; and music medicine, in which patients passively listen to recorded music under the guidance of medical staff^18^. In Vietnam, music intervention is a new concept, and most hospitals have not yet implemented it in any form. There is no academic course in music therapy in Vietnam, so the number of music therapists is limited. Thus, music medicine intervention that does not require a trained therapist is the only feasible strategy for psychological stress management in BGC patients in Vietnam.

Previous studies have mainly focused on evaluating stress levels and coping strategies using closed-ended questionnaires and have been limited to exploring the factors and situations that cause stress (stressors). Moreover, there is limited research on the implementation of music intervention for Vietnamese patients, especially cancer patients. Music is a product of culture and an aspect of cultural identity^19^. The wide variety of music in different cultures is guided by a set of five elements of music: rhythm, melody, pitch, harmony, and interval^20^. Good et al. suggested that nurses should be aware of the cultural differences in music preference and deliver culturally specific music selections to patients to achieve a therapeutic effect^21^. Additionally, to implement a new intervention in a population, the feasibility of the intervention should be considered, such as its potential acceptance, facilitators, barriers, and resource availability in the population^22^. Thus, cultural adaptation is necessary for nursing practice before implementing music interventions for stress management in BGC patients in Vietnam. The objectives of this study were as follows:

- To explore stressors perceived by BGC patients receiving chemotherapy during the pandemic.
- To compare the coping strategies of BGC patients receiving chemotherapy with those listed in the TMSC.
- To explore the cultural elements of music interventions among BGC patients receiving chemotherapy in Vietnam.
- To understand the feasibility of music interventions for BGC patients receiving chemotherapy in Vietnam.

## Method

An exploratory qualitative study with individual face-to-face semi-structured interviews was conducted. This study followed the Standards for Reporting Qualitative Research^23^ (see Supplementary File 1).

### Recruitment and sampling

A convenience sample of BGC patients receiving chemotherapy was recruited from the oncology centre of a public hospital in Nam Dinh, northern Vietnam. The patients’ data were collected from 30 June 2021 to 18 August 2021. The sample size was determined based on the data saturation principle^24^. The inclusion criteria were women who (1) had been diagnosed with BGC and were receiving chemotherapy, (2) were aged 18 and above, (3) had Karnofsky scores ≥ 60^25^, (4) had been informed about their cancer diagnosis, (5) could communicate in Vietnamese, and (6) consented to join the study. We excluded BGC patients who had severe psychiatric disorders or cognitive impairments.

A list of patients who were undergoing chemotherapy during the study period was obtained from an administrative nurse. The third author screened the health records of these patients for BGC diagnosis, age eligibility, and severe psychiatric disorders or cognitive impairments. Other criteria were confirmed by asking nurses who directly cared for the patients and by assessing the patients.

### Data collection

The third author approached eligible patients, invited them to participate in the study, and asked them to obtained signed consent forms from those who agreed to participate. The second author, who has experience in palliative care, individually interviewed all participants. Face-to-face interviews were conducted in a private room at the hospital, and most of the interviews lasted 40 to 45 min. A pilot interview was conducted on a patient to check the appropriateness of the interview questions and the patient’s understanding of the questions. The interviews were audio-recorded, and field notes were taken before, during, and after the interviews to note the information that could not be audio-recorded.

Fourteen open-ended questions were developed for the interviews based on the study objectives (see Supplementary File 2). The TMSC^7^ was used to develop an interview guide to explore the stress-causing factors and coping strategies among BGC patients receiving chemotherapy. Guidelines for music therapy practice in adult medical care^26^ were used to design questions to explore the patients’ perceptions of and preferences for music. In addition, two questions were designed to identify the potential facilitators of and barriers to implementing music interventions for BGC patients.

### Data analysis

The audio files were transcribed verbatim by the third author, and the first author listened to the audio recordings and cross-checked the transcripts to ensure accuracy. NVivo version 12 software was used for data management. Field notes and interview transcripts were analysed following qualitative content analysis as described by Graneheim and Lundman^27^. The first and second authors independently read the field notes and interview transcripts several times to obtain a sense of the whole^27^. The text was divided into meaning units, abstracted into condensed meaning units, and labelled with codes. The various codes were then compared to identify their differences and similarities and sorted into sub-categories and categories. Lastly, based on the fundamental meanings of the data, the categories were organised into themes^28^. The findings of the two authors were compared, and any disagreements were resolved through discussions with the last author.

### Trustworthiness

The trustworthiness of the study was ensured by evaluating the four criteria suggested by Lincoln^29^: credibility, dependability, confirmability, and transferability. The study was supervised by the last author, a professor with expertise in qualitative investigations^30–32^. The last author reviewed the study protocol before beginning the study and verified the study findings and interpretations. The other authors also have experience in qualitative research^33^. One author conducted all of the interviews following the interview guide to ensure consistency. The audit trail of data analysis, including the decision-making process and discussions among the authors, was documented. Member checking was performed to verify and correct the interview transcripts and ideas that extended beyond the original interview^28^.

### Ethical approval and consent to participate

All procedures performed in studies involving human participants were in accordance with the ethical standards of the institutional and/or national research committee and with the 1964 Helsinki Declaration and its later amendments or comparable ethical standards. The study was approved by the Ethics Committee of Survey and Behavioural Research Ethics, The Chinese University of Hong Kong (No. SBRE-20-813), and by the Ethics Committee in Medical Research, Nam Dinh University of Nursing, Vietnam (No. 1167/GCN-HDDD). Informed consent was obtained from all participants included in the study.

## Results

### Demographic characteristics of the participants

Twenty patients were interviewed, with ages ranging from 38 to 75 years. Most participants had below high-school-level education (80%), lived in a village (80%), and had stage III or stage IV BGC (60%). The characteristics of the participants are presented in Table 1.

**Table 1:** Demographic characteristics of the participants (N = 20).

Variables	n (%)
**Age (years) mean (standard deviation)**	
54.95 (10.83) (range, 38–75)	
**Education level**	
High school or above	4 (20%)
Under high school	16 (80%)
**Religion**	
None	12 (60%)
Buddhism	2 (10%)
Christianity	6 (30%)
**Current living place**	
City	4 (20%)
Village	16 (80%)
**Cancer stage**	
Stage I	2 (10%)
Stage II	6 (30%)
Stage III or IV	12 (60%)
**Treatment type**	
Chemotherapy only	9 (45%)
Chemotherapy plus radiotherapy	11 (55%)
**Chemotherapy cycle**	
1st, 2nd, or 3rd	11 (55%)
4th or above	9 (45%)

### Theme 1: stressors among BGC patients

Stressors are internal or external factors that cause psychological stress to individuals^7^. The patients underwent chemotherapy, yet each patient experienced stress from different stressors. The stressors identified for each patient are shown in Table 2.

**Table 2:** Stressors among patients with breast and gynaecological cancer.

Category	Subcategory
Undesirable experiences during treatment	Unexpected treatment results
	Uncertain future
	The restrictions imposed during the pandemic changed the patients’ treatment plan
Inability to fulfil their own roles and responsibilities	Being a burden to the family
	Inability to complete tasks

#### Undesirable experiences during treatment

During cancer treatment, undesirable outcomes, such as drug resistance or metastasis to other organs, can cause stress to patients: ‘*The first horrible experience was drug-resistant. Suddenly, my mind was a bit depressed*’ (P11). The patients’ feelings of uncertainty contributed to their stress. They did not know what would happen in the future. They were concerned about the progression of the illness, potential symptoms, or potential treatment-related adverse effects.

‘... also really worried; after the surgery was done, I was worried about chemotherapy. I didn’t know if it cost a lot of money or a little. Then I was also very worried. I didn’t know. After the surgery .... they gave me chemotherapy. I was also worried about how it works, whether my body could withstand it, .... I was very worried’ (P16).

Some included participants had been treated at Vietnam National Cancer Hospital in Hanoi, the most prominent cancer hospital in Vietnam, however, the surge of COVID-19 cases at the Vietnam National Cancer Hospital caused difficulty in using its services, especially for patients living outside of Hanoi. The patients reported that they could not visit the hospital because of the spread of the COVID-19 pandemic: ‘*… K hospital [Vietnam National Cancer Hospital] was locked down; I could not go. I stayed at home and didn’t go to the doctor. I was worried about my illness…’ (P19*). Then, they had to transfer treatment from the Vietnam National Cancer Hospital to Nam Dinh province hospital for continued treatment. Therefore, their treatment schedules had to change due to the pandemic.

‘I received one cycle of chemotherapy in Hanoi, but because of the pandemic I couldn't go for the next cycle. Then my illness worsened, and I had to come here [Nam Dinh hospital] to continue chemotherapy…’ (P11).

#### Inability to fulfil their own roles and responsibilities

The participants stated that their family members were worried about them and spent a lot of money on their cancer treatment. The participants perceived themselves as a financial burden on the family: ‘*Well, I just think that now that I’m sick. I’m causing suffering to both my husband and children, and the whole family has to worry’ (P2).*

The participants said that due to illness, they were unable to continue working or caring for their families. They worried that their children and families were not receiving enough care: *‘My most significant concern was about my child…because my child is still small. He is only six years old. If I get worse, what will happen to him…’ (P9).*

### Theme 2: coping strategies

Coping is defined as ‘constantly changing cognitive and behavioural efforts to manage specific external and internal demands that are appraised as taxing or exceeding the person’s resources’^7^. Emotion-focused and problem-focused coping strategies, as described in the TMSC^7^, were commonly adopted by the participants to reduce stress. Interestingly, a new emotion-focused coping strategy—self-realisation of responsibilities towards the family—was also identified (see Table 3).

**Table 3:** Coping strategies.

Category	Subcategory
Problem-focused coping	Use of instrumental support
Emotion-focused coping	Self-distraction
	Positive reframing
	Self-control
	Religion
	Use of emotional support
	Acceptance
	Self-realisation of responsibilities towards the family

#### Problem-focused coping

Only one problem-focused coping strategy from the TMSC^7^ was predominantly reported by the participants: the use of instrumental support.

Some patients actively sought advice from psychologists for their psychological stress: *‘I called and met a psychologist at K hospital. She gave me sleeping medicine. She said that I had to sleep properly for my illness to heal quickly…’ (P17).*

To improve their knowledge of the disease and its treatment, they actively read information about cancer treatment: *‘Sometimes I research which fruits and vegetables are good to eat…I do research about foods for cancer as well’* (P1).

#### Emotion-focused coping

The participants adopted a range of emotion-focused coping strategies to manage stress, most of which were in line with the TMSC^7^, namely self-distraction, positive reframing, self-control, religion, use of emotional support, and acceptance.

Many participants said that they engaged in other activities, such as shopping, housekeeping, watching movies, listening to music, and engaging in other forms of entertainment, to reduce their emotional distress: *‘Since becoming sick, I often listen to music, often turn on meditation music for me to listen to. I think the meditation music is calming, and it helps me to relieve stress’ (P13)*.

Some participants thought that cancer was their fate, so they believed they had to accept it. However, others achieved stress reduction by finding the positive in adverse events; that is, they ‘looked for the silver lining or tried to look on the bright side of things’^7^. They may have known numerous BGC survivors who had lived a very long time, or they may have considered the fact that they had the mildest and most curable type of cancer: *‘I have breast cancer, which is still lucky because it is milder than other cancers. People with stomach cancer can’t even eat, so they need many people to take care of them’ (P12).*

The participants found that engaging in religious activities, such as prayer, Buddhist chanting, or listening to the pastor’s preaching, helped them feel less stressed and anxious: *‘Many nights when I couldn’t sleep, I prayed to Buddha and asked him to help me’ (P15)*. Most participants reported seeking emotional support from family, medical staff, friends, and other cancer patients to overcome this stressful period in their lives. The spiritual support from family members, especially the husband, was mentioned by many participants.

‘My husband and children gave me courage .... in this hospital, I was taken care of and inspired by the doctors. When I returned home, my husband and children or my brothers and relatives also came to give me spiritual encouragement to make me comfortable...’ (p7).

Self-realisation of responsibilities towards the family is explained by the following cognitive process: when under stress from a significant health threat, the individual recognises their worth and their obligations towards their family and society. This provides them with increased self-assurance and stamina to handle challenging circumstances. Our participants cited their children, husbands, and families as important sources of motivation to overcome difficult circumstances. Thinking of their children and realising their responsibilities towards their families motivated the participants to continue fighting their illness: *‘I tried to live for my children…now everything I do is for my children. If I can control it, my children can be helped…’ (P13).*

### Musical preferences

Table 4 lists the three music genres most preferred by the participants—religious music, softly melodic music, and revolutionary music—and the two most disliked music genres—fast, intense music and ancient folk songs with languid melodies.

**Table 4:** Preferred and non-preferred music genres.

Themes	Category	Subcategory
Preferred music	Religious music	Buddhist music
		Christian music
	Softly melodic music	Folk songs
		Relaxing instrumental music
		Vietnamese bolero
	Revolutionary music	
Non-preferred music	Fast, intense music	Rock
		Rap
		Electronic music
	Ancient folk songs with languid melodies	Chèo
		Tuồng

#### Preferred music genres

Some participants said that they only listened to religious music, such as Buddhist and Christian music. Buddhist music is created to communicate communal joys, excitements, and sorrows^34^ as well as to honour the Buddha, encourage Buddhist piety, and convey the Buddha’s teachings to help people live meaningful lives^35^. Buddhist music has a polyphonic texture generated by subtly vibrating aural chords. In addition to serving as a way of bringing the petitions of sentient beings to the Buddha, bells, mufflers, chanting, and the ethereal light of candles and incense smoke used during prayers also have the power to awaken the goodness of people’s nature, reminding them of their Buddhist values^34^. Modern Vietnamese Buddhist music also contains pieces with mellow, soothing melodies and slow beats that can incorporate contemporary musical elements, such as pop ballads, and Vietnamese traditional musical elements to promote relaxation.

Regarding Christian music, its lyrics typically address issues about the Christian faith. Before 1980, Vietnamese Christian music used Western hymns translated into Vietnamese. Since the August Revolution of 1945, Vietnam has seen a cultural transformation, including a revolution in musical compositions and hymns. Vietnamese Christian music has undergone many changes to accommodate the local culture. Since 1980, Vietnamese Christian music has frequently incorporated local folk tunes and lyrics. For instance, Northern Vietnamese Christian music often contains Quan Ho folk songs, and Southern Vietnamese Christian music contains Southern Vietnamese and Cai Luong folk songs. Modern Vietnamese Christian music also uses modern instruments, such as the organ, together with the traditional ethnic musical instruments, such as the flute and đàn nhị^36^.

‘... I am Christian, so I often listen to Christian music. Sometimes, when I am down and feel sad, I open myself up to listen. I listen to Christian music a lot. I don’t listen to any other music except Christian music ...’ (P7)

Most participants also reported a preference for softly melodic music, including Vietnamese folk songs, relaxing instrumental music, and Vietnamese bolero. Originating in Spain, bolero was first introduced to Latin America and then to Vietnam in the early 1950s. An important characteristic of bolero music is that it can easily be adapted to Vietnamese culture, making it easy for Vietnamese people to sing and memorise. Most Vietnamese bolero songs are influenced by Southern folk songs and thus have a simple melody and a regular, slow, 4/4 time rhythm, with little variation in tempo and few high ranges.

Some participants especially preferred music with revolutionary themes written during the Vietnamese War. Vietnamese revolutionary songs often aimed to promote the fighting spirit of soldiers, communicate state policies, and encourage love for communist and socialist ideals. In addition, revolutionary lyrical songs express love for the homeland or promote labour, construction, optimism, love of life, dedication, and community. Vietnamese revolutionary songs are mostly chamber music with folk lyrics sung by a tenor and soprano or choir. They have complex melodies but simple lyrics to reach the masses. Excluding marches, suites, and choral pieces, which are often sung in groups, most revolutionary songs are sung in a bright, high, and wide-ranging major key, sometimes accompanied by a chorus. Revolutionary music often incorporates marches, valses, slow waltzes or the Boston, slow ballads, slow surf music, blues, chachacha, disco, and complex melodies^37^.

#### Non-preferred music

Many participants reported that music with a fast beat and intense, powerful styles, such as heavy metal, rap, or electronic music, normally preferred by the young generations, did not interest them. Some participants even reported getting headaches and feeling irritated by such music. Although some participants reported their affinity for the traditional musical forms of Chèo and Tuồng, many expressed that their melodies were too slow and unpleasant. Chèo is more instantly accessible because it involves melodic and percussive instruments and appealing rhythms and is frequently accompanied by dramatic stage performances by dancers^38^. Hexasyllabic and octasyllabic pairs of verses make up the majority of lyrics. The melodic and rhythmic patterns can be changed using peg words with extra syllables^39^. Chèo’s music essentially depicts the routine and straightforward existence of farmers in rural areas^40^.

Tuồng focuses on praising the heroic actions of the nobility and elites in society^40^. With its use of colour, attire, cosmetics, gesture, and movement standards, Tuồng is a highly symbolic form of musical theatre involving a scholarly or civilian female character depicted by a person pointing with one finger and a martial character depicted by a person pointing with two fingers. The gestures and actions in the theatrical performance of this music are also very symbolic. The Confucian qualities of loyalty, righteousness, justice, and filial piety are frequently included in the legendary, historical, and contemporary narratives presented in Tuồng. A Tuồng performing group includes a percussionist who plays various drums and is regarded as the leader. The kèn double-reed oboe, one of the loudest instruments in the Tuồng ensemble, has the most noticeable sound, a piercing nasal tone, of all instruments in the ensemble. Tuồng also involves various vocalisations, such as Nói Thường, which is ordinary speech without any discernible poetic meter; Nói Lối, which is stylised speaking in a higher pitch with some sense of rhythm because the text is in rhythmic couplets; Hát Nam, which is thought to be influenced by songs from the Cham ethnic minority; and Hát Khách, a singing style thought to be influenced by Chinese music due to its use of the pentatonic scale. Some other styles of vocalisation are only adopted for very dramatic performances^41^.

‘The rock and rap genres don’t suit me. I don’t like listening to those genres. At home when anyone is listening to rock or rap music, I don’t like it…’ (P14).

‘I hate Chèo the most. It is too slow for my liking … I hate it [laugh]’ (P15).

#### Perception of music

The participants’ perceptions of music interventions were divided into two categories: perceived beneficial effects and perceived individual differences in musical preferences among the participants. The categories and sub-categories are illustrated in Table 5.

**Table 5:** Perception of music.

Category	Subcategory
Perceived beneficial effects	Enjoyment
	Relaxation
	Distraction from unpleasant experiences
Perceived differences in musical preferences of individuals	Musical preference is different between generations
	Musical preference is different between religions

#### Perceived beneficial effects

Most patients reported that music could lift their mood, provide inner peace, reduce their stress, and help them think more optimistically.

‘When I listen to music, my mind feels relaxed; it helps me forget my illnesses and sadness. I immerse my soul in the music. It’s very pleasant’ (P7).

#### Perceived individual differences in musical preference

Several patients thought that music preference differs by generation: *‘The elderly listen to the Buddhist songs…but the young generation is different’ (P18).* Participants believed that musical preference differs by religion: *‘…each religion is different. I am Christian, I sing Christian songs, but people of other religions in society listen to different songs’* (P21).

### Acceptance of music interventions

Listening to music was a hobby for many participants. Many participants believed that music therapy could be beneficial but expressed their preference for listening to the music genres they loved (see Table 6).

‘If I can listen to music that suits me, my favourite music, I will participate’ (p5).

‘I’d love to participate. If you could give me my Christian music, I'd love it more’ (P21).

**Table 6:** Acceptance of music interventions.

Category	Subcategory
Accepted music interventions	Listening to music as a hobby
	Listening to preferred music genres
Rejected music interventions	Not like music

However, several patients who did not enjoy listening to music were reluctant to participate in music interventions: *‘Listening to music is not my hobby’ (P4).*

### Facilitators and barriers to implementing music interventions

One category of facilitators (‘being supported’) and two categories of barriers (external and internal factors) to implementing music interventions were identified (see Table 7).

**Table 7:** Facilitators and barriers to implementing music interventions.

Themes	Category	Subcategory
**Facilitators**	Being supported	Having companions to listen to music with
		Having various devices on which to listen to music
**Barriers**	External factors	Lack of devices or the Internet
		Lack of private environment
	Internal factors	Low confidence in using technology

#### Facilitators

Some participants said that their husbands also liked music and that they often listened to music and sang together. Some participants with similar musical preferences also regularly listened to music and sang together in the hospital. Many participants reported that they had several devices on which to listen to music, such as smartphones and smart TVs at home.

‘Both my husband and I love to listen to music’ (P3).

‘At the hospital, a few of us sang together. With people who love the same music, it’s fun’ (P20).

‘At home, I have too many devices. I also have a phone like yours [pointing to a smartphone]. I don’t need a sim card, but I can watch whatever I want’ (p15).

#### Barriers

Many participants either lacked the necessary devices or refused to carry expensive devices to the hospital. Others complained that they were unable to use the Internet at the hospital: *‘There is no Internet here [hospital]’ (P17)*. Some participants, especially older adults, expressed challenges in using technology to play music: *‘I'm old, so I rarely listen to music because I can’t use my phone’ (P11)*. Some participants said that they wanted to listen to music, but the room was so crowded with patients that they did not want to disturb them: *‘When I’m being treated at the hospital, the hospital is very crowded. Not every patient has a bed, and sometimes, two patients share a bed. Maybe I like listening to music, but other people don’t want to listen to it. So, I avoid listening to not disturb other people in the room’ (P2).*

## Discussion

This study identified the common stressors that caused stress to BGC patients receiving chemotherapy during the COVID-19 pandemic. The results showed that cancer patients experienced a variety of stressful situations. A new emotion-focused coping strategy—self-realisation of responsibilities towards the family—was identified in this population, in addition to the coping strategies described in the TMSC. This is a promising coping strategy that should be implemented and explored in future stress management programmes. This study also explored musical preferences and music perception as the main cultural elements of music interventions among BGC patients receiving chemotherapy in Vietnam. Acceptance of music interventions and facilitators of and barriers to implementing music interventions were also investigated, and the findings are expected to guide the implementation of future music interventions.

The COVID-19 pandemic has hampered cancer patients’ access to medical facilities for chemotherapy, thereby contributing to their stress and worry about their cancer progression and treatment effects. Our results are consistent with a previous study in the UK that reported that the lockdown during the pandemic disrupted cancer care services and increased anxiety, depression, and cognitive dysfunction in breast cancer patients^4^.

In our study, problem-focused and emotion-focused coping strategies, in line with the TMSC, were commonly reported by the participants. Most of the reported sub-categories of coping strategies were also consistent with the TMSC. However, we also identified a new emotion-focused coping strategy: self-realisation of responsibilities towards the family. Several participants emphasised that family was a major source of emotional support for stress management. This is consistent with a study by Ozdemir and Tas Arslan^42^ that confirmed that family support plays a crucial role in stress management.

The family also functioned as a coping resource for the participants in another way: it made these women living with BGC aware of their obligations as wives or mothers. These roles and responsibilities prevented them from feeling weak and giving up. Vietnamese women are known for their immense devotion to and sacrifice for their families. They are willing to devote their entire lives for the well-being of their cherished offspring^43^. Therefore, even when suffering from a serious disease or facing death, they maintain strength and courage owing to the feeling of responsibility towards their families. In this study, the participants’ most common worry during their poor health was that their families and children would not be well cared for. Thus, their families and children were a source of strength and determination for them to fight cancer and overcome difficult and stressful situations.

The BGC patients in this study were aware of the benefits of music for psychological health. For many participants, listening to music had become an indispensable spiritual aspect of life and was used to overcome stressful situations. This suggests that music interventions could be effective in reducing stress of BGC patients in Vietnam. However, the participants expressed that the music intervention would be more acceptable and effective if they were allowed to choose their preferred genre of music to listen to^44^. Respecting patients’ musical choices might help to prevent side effects of listening to non-preferred music genres, such as headaches and irritation, which were reported by some participants.

Many participants also took pleasure in listening to folk music, religious music, or music with soothing melodies that had themes of love and patriotism. As Vietnam has experienced prolonged and brutal wars, songs with a national and revolutionary spirit focusing on the military and war heroes are favoured by many older citizens. In our study, most of the participants were middle-aged and older adults who had personally experienced the war or the challenging conditions following the war, which explains why revolutionary music had become deeply ingrained in their lives.

## Limitation and implications

There are several limitations to this study. First, we only interviewed BGC patients at one hospital. As musical preferences may differ regionally, further studies in other regions of Vietnam are warranted before music interventions could be implemented for BGC patients. Second, because of the restrictions during the COVID-19 pandemic, a limited number of eligible patients were available to participate in the study. Thus, not many patients with distinct characteristics could be included.

However, our findings have important implications for future research and practice. Patients may experience psychological stress at any time during the cancer treatment trajectory due to undesirable or adverse effects of cancer treatment. During the ongoing pandemic, which adds to the stress levels of cancer patients, it is important to re-evaluate effective coping strategies for stress management^45^, as done in the present study. Our findings highlight the importance of patients’ awareness of their values, roles, and responsibilities towards their children and families, in addition to family support^7,46,47^, to promote stress management. This self-awareness process may occur differently in each individual. To promote this process in BGC patients and subjects with severe health problems, psychotherapy, such as dignity therapy, could be administered to help them cope with stressful situations. For example, a therapist or health professional could ask questions or engage in discussions to recall the patient’s life history, achievements, significant roles, hopes, and dreams, all of which could be sources of strength and motivation to overcome stress. Further studies are needed to identify more coping strategies for different patient populations facing specific stressful situations. Notably, music is an inexpensive, safe, non-invasive intervention for stress reduction^48^ that can be easily implemented for BGC patients undergoing chemotherapy. Importantly, music interventions that allow patients to listen to their preferred music genre will be readily acceptable in the population. Nurses could pre-select a list of music pieces in patients’ preferred genres, as they are more likely to have a therapeutic effect than pieces in non-preferred genres. Nevertheless, other factors such as hearing ability and musical anhedonia need to be taken into account before implementing music interventions for cancer patients.

## Conclusion

BGC patients undergoing chemotherapy have been experiencing high levels of stress during the ongoing COVID-19 pandemic. We identified a new emotion-focused coping strategy—self-realisation of responsibilities towards the family—that was effective in lowering the patients’ stress levels. The study results further support the feasibility of music interventions for reducing stress levels of BGC patients in Vietnam. The study also provides a list of preferred musical genres for stress management interventions and the common facilitators of and barriers to implementing such interventions.

## Supplementary Information

Supplementary Information 1.

Supplementary Information 2.

## Data Availability

All data generated or analysed during this study are included in this published article.
