# Supplementary material for: A qualitative study on stress, coping strategies and feasibility of music intervention among women with cancer receiving chemotherapy during COVID-19 pandemic in Vietnam
Source: Sci Rep. 2023 Jan 11;13:542. doi: 10.1038/s41598-023-27654-9 (PMC9832410; doi:10.1038/s41598-023-27654-9)
Supplement: Supplementary file 2 — Supplementary Information 2. [file 41598_2023_27654_MOESM2_ESM.docx]

**Appendix A: Interview guide**

1. How is your emotion recently?
2. Can you describe your emotional experiences during chemotherapy treatment?
3. What is your stress level during chemotherapy treatment?
4. How did you do to cope with stress during cancer treatment?
5. How was your family, friends and medical staff supporting you?
6. How does music influence your life?
7. What types of music do you listen to for pleasure?
8. Is there a particular style of music that you find relaxing?
9. Is/are there particular artist(s) or song(s) that you enjoy listening to?
10. Are there any types of music or music selections you Do Not like?
11. What do you think about music as a means of stress management?
12. What do you think if we offer music to you undergoing chemotherapy?
13. What will be the difficulties for you in listening to music?
14. What are the difficulties and facilitators to maintaining music listening at your home?
